# Global Prediction of Tissue-Specific Gene Expression and Context-Dependent Gene Networks in *Caenorhabditis elegans*

**DOI:** 10.1371/journal.pcbi.1000417

**Published:** 2009-06-19

**Authors:** Maria D. Chikina, Curtis Huttenhower, Coleen T. Murphy, Olga G. Troyanskaya

**Affiliations:** 1 Department of Molecular Biology, Princeton University, Princeton, New Jersey, United States of America; 2 Lewis-Sigler Institute for Integrative Genomics, Princeton University, Princeton, New Jersey, United States of America; 3 Department of Computer Science, Princeton University, Princeton, New Jersey, United States of America; Washington University, United States of America

## Abstract

Tissue-specific gene expression plays a fundamental role in metazoan biology and is an important aspect of many complex diseases. Nevertheless, an organism-wide map of tissue-specific expression remains elusive due to difficulty in obtaining these data experimentally. Here, we leveraged existing whole-animal *Caenorhabditis elegans* microarray data representing diverse conditions and developmental stages to generate accurate predictions of tissue-specific gene expression and experimentally validated these predictions. These patterns of tissue-specific expression are more accurate than existing high-throughput experimental studies for nearly all tissues; they also complement existing experiments by addressing tissue-specific expression present at particular developmental stages and in small tissues. We used these predictions to address several experimentally challenging questions, including the identification of tissue-specific transcriptional motifs and the discovery of potential miRNA regulation specific to particular tissues. We also investigate the role of tissue context in gene function through tissue-specific functional interaction networks. To our knowledge, this is the first study producing high-accuracy predictions of tissue-specific expression and interactions for a metazoan organism based on whole-animal data.

## Author Summary

In animals, a crucial facet of any gene's function is the tissue or cell type in which that gene is expressed and the proteins that it interacts with in that cell. However, genome-wide identification of expression across the multitude of tissues of varying size and complexity is difficult to achieve experimentally. In this paper, we show that microararray data collected from whole animals can be analyzed to yield high-quality predictions of tissue-specific expression. These predictions are of better or comparable accuracy to tissue-specific expression determined from high-throughput experiments. Our results provide a global view of tissue-specific expression in *Caenorhabditis elegans*, allowing us to address the question of how expression patterns are regulated and to analyze how the functions of genes that are expressed in several tissues are influenced by the cellular context.

## Introduction

Tissue-specific gene expression is a fundamental aspect of multicellular biology, underlying the development, function, and maintenance of diverse cell types within an organism. Accounting for tissue-specific expression is a precursor to any systems-level understanding of metazoan organismal development and function and large-scale studies of spatio-temporal gene expression both at the single-gene and whole-genome level have been performed in several organisms [1]–[5]. Additionally, tissue specificity is an important aspect of many complex diseases; notable examples of tissue interactions associated with disease include stroma-tumor interactions in cancer [6] and tissue-specific effects of insulin signaling in diabetes [7]. Although several experimental techniques have been developed to identify tissue-specific gene expression signatures, both at the single-gene and whole-genome level, our current knowledge of tissue-specific expression is incomplete.

The model organism *Caenorhabditis elegans* provides a good framework for the study of tissue-specific expression. Its invariant cell lineage allows single-cell resolution of tissue-specific expression patterns through a variety of experimental techniques [5],[8]. *In situ* hybridizations of the entire transcriptome are in progress [9], and GFP-promoter tagging has been applied on a large scale [8],[10],[11]; as a result, the expression of approximately 3500 genes has been studied at the single-gene level [12], providing a “gold standard” for gene expression. Additionally, several methods have been developed to isolate mRNA samples enriched for a specific tissue or cell type, allowing global analysis using microarrays or SAGE [13]–[22].

Despite the variety of techniques available and the number of studies performed thus far, our understanding of tissue-specific expression in *C. elegans* is not yet complete; most genes have not been analyzed at the single-gene level, nor under diverse conditions and developmental stages. Additionally, each of the individual techniques for measuring tissue-specific expression suffers from drawbacks. GFP-promoter constructs, though they present the most accurate method amenable to high-throughput analysis, may incompletely capture endogenous expression or may fail to express well, a problem that is particularly severe in the germ line due to silencing [23]. Directed microarray studies, while powerful, depend on the ability to isolate mRNA from a particular tissue, since dissection is not possible in most cases, and methods to achieve this each have disadvantages: studies using mutants may report non-endogenous expression; embryonic cell sorting misses expression that only occurs in later stages of development, as post-embryonic cell sorting is not yet feasible; and poly-A binding studies depend on the ability to introduce the binding protein construct into and extract the protein out of the tissue of interest [21]. Thus, the ability to directly study the expression specificity of each gene across tissues, especially small tissues, and ideally to also account for the effects of development and environmental conditions, remains challenging.

Here we present a computational method that leverages existing experimental information to expand and improve our knowledge of tissue-specific expression. Using data from whole-animal microarrays, we accurately predict tissue-specific expression in all major tissues and even for several tissues that comprise only a few cells. Our approach not only outperforms directed high-throughput studies in all but one case, but also captures information that complements existing experiments, for example, by uncovering tissue-specific expression that is only seen under specific conditions. To confirm our predictions, we experimentally verified the expression of several genes. We have made our predictions available through a dynamic web-based interface at http://function.princeton.edu/worm_tissue to enable hypothesis generation and further experimental follow up by the community.

Using this accurate large-scale, tissue-specific information, we perform further computational analyses, such as prediction of transcriptional regulatory motifs specific to understudied tissues as well as tissue-specific miRNA target regulation. In addition, we extended our algorithm to produce tissue-specific functional interaction networks that provide a framework for discovering protein function specific to particular tissues. Our ability to uncover tissue-specific information should allow higher-detail analysis of expression and further hypothesis testing to identify expression changes that are important for biological function.

## Results

### Tissue-specific signals in whole-animal microarrays

**Figure 1:**
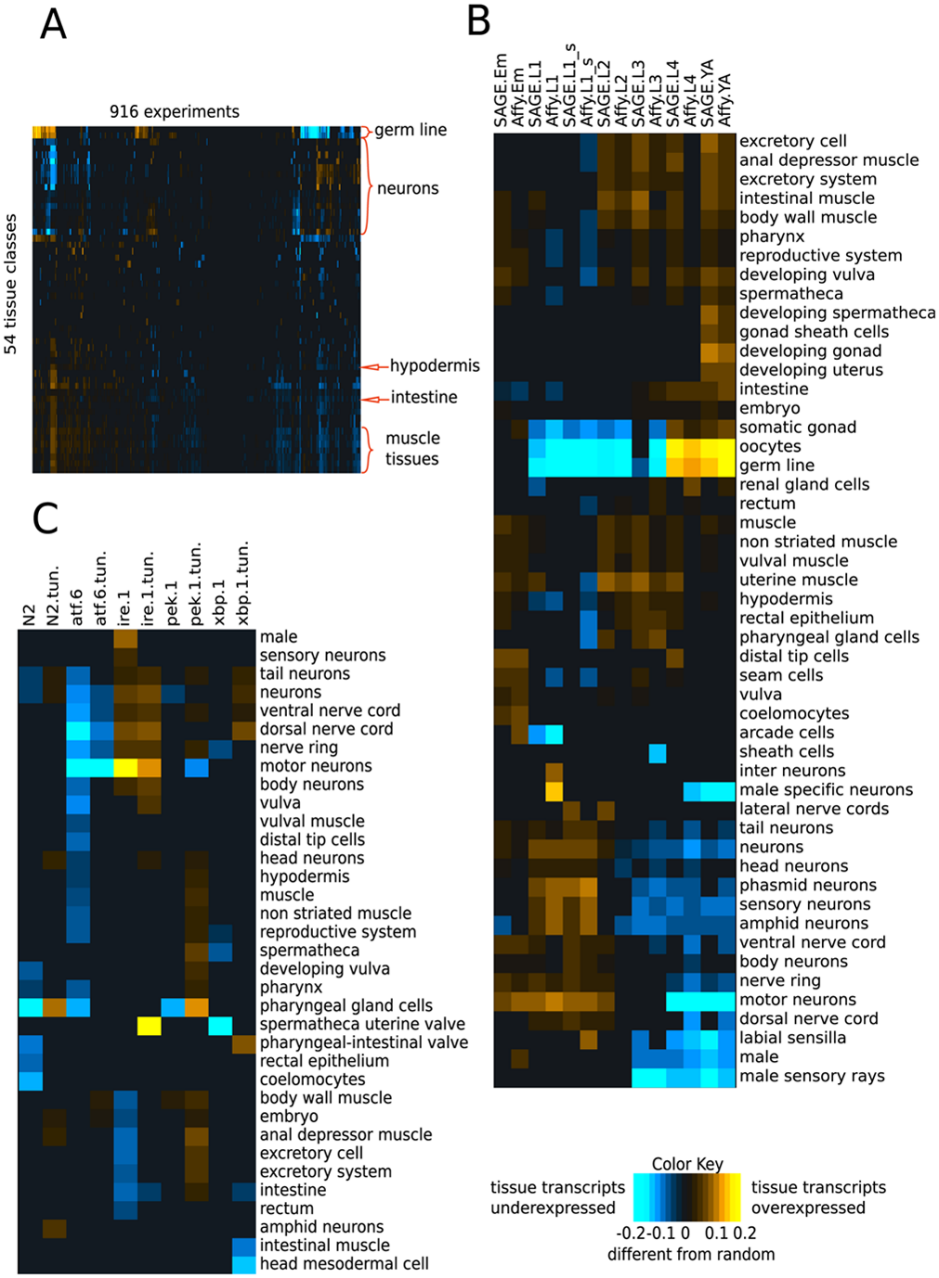
Over- or under-expression of tissue-specific transcripts is quantified using a rank-sum statistic corrected to 0.05 false discovery rate. Tissues with no significant results are omitted. (A) A global view of all microarray experiments (clustered on both axes). Significant tissue biases can be observed across the compendium, with neuronal and germ line signals being especially prevalent. Yellow square for a specific tissue and condition combination indicates that genes known (in our gold standard, see Methods) to be expressed in that tissue are over-expressed -(as compared to background) in that microarray condition. (B, C) Detailed views of parts of the matrix in (A). (B) Levels of over- or under-expression of tissue-specific transcripts in developmental time course experiments on SAGE and Affymetrix platforms [24] (clustered on both axes). Over- and under-expression of tissue-specific genes coincides with the timing of tissue development. (C) Levels of over- or under-expression of tissue-specific transcripts in the unfolded protein response study Shen et al. [25] (clustered on the y axis). Mutations in the UPR pathway genes invoke tissue-specific responses. Treatment with tunicamycin is denoted as (tun.).

We compiled a large compendium of *C. elegans* microarray data (comprised of 916 experiments from 53 datasets). A few (16) of these microarray studies address tissue-specific expression, but most studies examined changes in gene expression in the animal as a whole (see supplementary website at http://function.princeton.edu/worm_tissue for a list of microarray experiments used). Using a rank-based statistic, we evaluated the level of under- or over-expression of genes associated with each tissue in a given microarray experiment against a “gold standard” of 2872 genes known to be expressed in a particular tissue. Our gold standard is composed of information derived from single gene studies such as promoter-GFP tagging, antibody staining, and *in situ* hybridizations (WormBase), which we hand curated to account for tissue naming synonyms. The gold standard also includes the 1872 promoter –GFP fusions from the *C. elegans* Tissue Expression Consortium [10],[11],[24]. Importantly, the gold standard is completely independent from the microarray or SAGE gene expression data in our compendium. This gold standard of tissue-specific gene expression allowed us to identify substantial tissue bias in the transcriptional responses of microarray experiments. We quantified over or under-expression of tissue-specific gene sets using a rank-based statistic (Figure 1A). Despite the fact that only a small number of studies isolated specific tissues, we found that tissue-specific signals can be observed in many whole-animal experiments. For example, analysis of two developmental time courses [24] revealed dramatic tissue-specific temporal patterns that reflect developmental timing; as might be expected because neurons are born in early larval stages, earlier developmental stages are enriched for neuronal transcripts, while later stages are enriched for germ line transcripts, correlating with the development of reproductive tissues and the onset of reproduction (Figure 1B). We can also quantify a number of previously uncharacterized tissue-specific responses. For example, motor and sensory neurons have distinct developmental profiles (Figure 1B) and, in contrast to other non-reproductive somatic tissues, intestinal expression steadily increases with developmental stage.

Tissue-specific responses can also be observed when experimental treatments are applied to animals in the same developmental stage. For example, our analysis of tissue-specific signals in a whole-animal microarray study of unfolded protein response [25] revealed that various mutations in UPR pathway genes have different effects on tissue-specific expression (Figure 1C). Consistent with previous studies [26],[27], we observed that an *ire-1* mutation has a strong effect on epithelial tissues such as the intestine and the excretory cell, as genes expressed in those tissues are significantly down-regulated as a result of *ire-1* mutation. On the other hand, an *atf-6* mutation causes a decrease in neuronal transcripts suggesting greater reliance on the *atf-6* branch of the UPR in neurons. Distinct tissue-specific profiles can be observed for other treatments as well. Thus, our analysis demonstrated that we can identify both known and novel tissue-specific expression information from existing gene expression microarray experiments.

### A computational method to accurately predict tissue-specific expression

**Figure 2:**
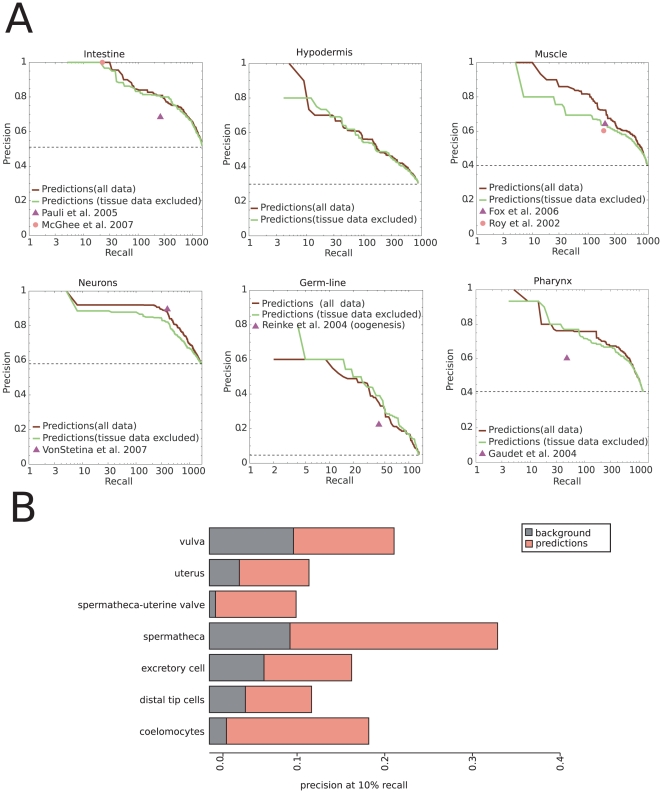
Classifier performance. (A) Accuracy of predictions for major tissues. These precision-recall plots demonstrate the trade-off between the number of genes predicted and the fraction of the predictions that is correct. Red lines show the performance of our approach using all available microarray data, while green lines show performance using only whole-animal studies. High precision can be achieved by using whole-animal experiments alone. The accuracy and coverage of existing high-throughput studies is shown with triangles and circles. The use of datasets addressing tissue-specific expression improves accuracy in some but not all cases. (B) Precision at 10% recall for small tissues compared with expected precision based on the genomic background. We compare the fraction of true positives in the top 10% of our predictions against the fractions that would be expected if the genes were chosen randomly (genome background).

The previous examples suggest that substantial information about tissue-specific expression can be gained by a directed analysis of whole-animal microarray data. As such, we applied a state-of-the-art machine learning algorithm, support vector machines (SVM) [28], to build a predictive model of tissue-specific microarray profiles. Intuitively, SVM automatically identifies expression patterns in our compendium whose combination maximally separates genes expressed in a particular tissue (e.g., neurons) from other (e.g., non-neuronal) genes. This classifier can locate hidden tissue-specific expression patterns that are scattered through only a few experiments in the compendium and might come from diverse types of studies. By contrast, clustering methods (e.g. standard hierarchical clustering [29] or the *C. elegans* TopoMap [30]), while clearly important for functional data exploration, cannot detect these signals at resolution sufficient for prediction of tissue-specific expression (see Table S1 for comparison between correlation and SVM). Using the SVM classifier to predict tissue-specific gene expression based on the microarray compendium, we achieved a high degree of accuracy, outperforming directed microarray-based studies of tissue-specific expression in most cases. Our evaluation is based on the standard cross-validation technique, where only a fraction of the genes with known expression is used for building the classifier while the rest is held out for evaluation. Our predictions reach a precision of 90% for all of the major tissues of the worm (intestine, hypodermis, muscle, neurons, and pharynx) except germ line (Figure 2A). It is likely that germ-line performance is substantially underestimated, since the expression of many of the genes in the gold standard was investigated using promoter-GFP fusions, which are often germ-line silenced [23].

We also evaluated performance of other tissue-enriched gene lists acquired from directed microarray experiments against the same gold standard, using the processed lists from each publication. In all but one case, our approach outperforms these studies, predicting more genes at higher accuracy (Figure 2A). The single exception is the neuronal gene list published by Von Stetina *et al.* [22] that correctly recalls 384 of our gold standard neuronal genes with 89% accuracy, somewhat above what we are able to predict at the same recall.

Our method accurately predicts tissue-specific gene expression even from whole-animal microarray data alone. When we exclude the 16 studies that directly address tissue-specific expression, our prediction accuracy remains high; in some cases it is even unchanged (Figure 2A, “tissue data excluded”). In particular, even for the intestine, for which there are a number of high-quality directed studies [17],[18], our prediction accuracy is not decreased when we use only whole-animal data.

Functional analysis of the top tissue-specific predictions (GO enrichment analysis) demonstrates that many of the genes we predict to express in specific tissues have functions consistent with that tissue. For example, predictions for germ line expression were enriched for cell cycle-related GO terms, those for muscle included “muscle contraction” and “respiration”, intestine included terms related to digestion and metabolism such as “fatty acid biosynthetic process”, neuron predictions were associated with “synaptic transmission” and “memory”, and hypodermis-expressed predictions included enrichment for terms related to molting and cuticle components. The pharynx is a complex organ that is comprised of muscle, structural and gland cells and genes predicted to express in the pharynx are enriched for diverse functions related to cytoskeleton, cuticle components, and secretion. (See supplementary website for all GO enrichment results.)

### Predicting tissue-specific expression for smaller tissues

While techniques for isolating tissue-specific mRNA are steadily improving, it remains a particular challenge to examine the expression of genes in smaller tissues. Therefore, it is of particular interest to be able to predict expression in tissues that are comprised of only a few cells. Using our approach, we were able make high-quality predictions for many tissues where biochemical methods have yet not been successfully applied. While we do not achieve the high level of precision we observe in major tissues (which is expected, as far fewer genes are reported to express in the smaller tissues, making new candidates significantly more difficult to identify), we were able to identify genes that are significantly enriched for expression in the small tissue of interest when compared to the genomic background (Figure 2B). For example, among the genes in our gold standard, only 1 in 10 express in the vulva. However, we were able to correctly recall 30% of all vulval genes with a precision of 20% percent, a two-fold improvement above the genomic background rate, and likely an under-estimate as our GFP-based gold standard is far more incomplete for these small tissues than for larger tissues. Among the genes that scored highly in vulval predictions is *dgn-1*, a homolog of human dystroglycan. The gene was not a top prediction for any major tissue except pharynx, suggesting that it is not widely expressed. Among small tissues, *dgn-1* was predicted to express in the uterus, distal tip cells, and the excretory cell in addition to the vulva. While *dgn-1* was not included in our gold standard, expression in these tissues, including expression in pharyngeal epithelia, has been confirmed recently [31]. Additionally, this gene has been shown to be functionally important for the development of the vulva and the excretory cell [31],[32], in contrast to its vertebrate homolog, which functions in muscle.

Among other small tissues, we were also able to make reasonable predictions for the excretory cell, the spermatheca, the uterus, ceolomocytes, and distal tip cells. In many cases the predicted genes have annotations that are consistent with the function of the tissue. For example, our distal tip cell predictions are enriched for many GO terms including “cell migration,” “protein localization,” and several “cellular component” terms associated with exocytosis. These GO associations appear reasonable, as distal tip cells are two highly polarized cells that lead gonad migration during development. Secretion from these cells is known to play an active role in gonad migration [33], and the cells' morphology (as visualized by EM) is indicative of active endo/exocytosis [34]. In addition, the top 200 distal-tip cell predictions significantly (*p*<10^−2^, hyper-geometric test) overlap with the list of genes associated with distal tip cell migration phenotypes compiled in a recent RNAi study [35]. Thus, our results demonstrate that even small tissues that are challenging to isolate experimentally have distinct expression profiles within whole-animal microarray data. Our ability to make such predictions will likely improve as new gene expression experiments are added to the compendium.

### *In vivo* validation of predicted genes

**Figure 3:**
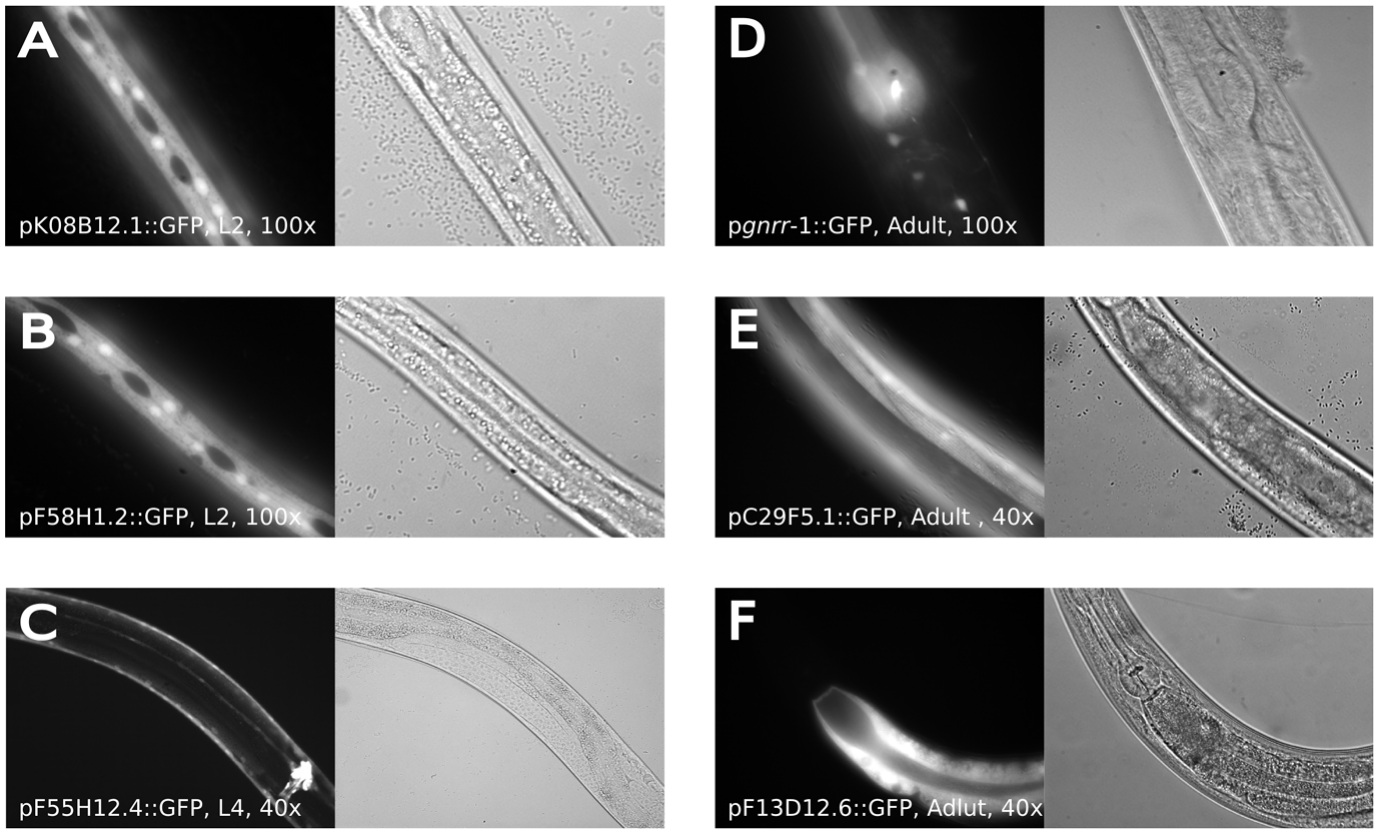
Expression of GFP-reporter constructs. (A) K08B12.1 was predicted to express in hypodermis; the reporter construct expressed exclusively in hypodermis. Expression was variable, strongest in embryo-L1, though detectable in all stages. (B) F58H1.2 was predicted to express in hypodermis; the reporter construct expressed exclusively in hypodermis. Expression was variable, strongest in embryo-L1 and not detectable in adults. (C) F55H12.4 was predicted to express in the hypodermis. pF55H12.4::GFP expressed in hypodermis, vulva, anus, and to a lesser extent pharynx. Hypodermal expression was highly variable and was strongest in L4 and Adult stages. (D) C29F5.1 was predicted to express in muscle. The reporter construct was observed in body wall, vulval, and anal but not pharyngeal muscle in all stages. (E) F13D12.6 was predicted to express in the intestine and the reporter construct expressed exclusively in intestinal cells at all stages. (F) *gnrr-1* was predicted to express in neurons. Strong expression of p*gnrr-1*::GFP was seen in various head neurons at all stages. Expression was also observed in the anterior pharynx and ventral nerve cord neurons. (A,B) Seam cell exclusion is observed in these lines, which is typical of hypodermally expressed genes; see Gilleard et al. [77] for examples of hypodermal expression.

We experimentally verified tissue-specific expression of six top genes with previously unreported tissue-specific predictions by creating transgenic lines carrying promoter-GFP constructs (Figure 3). Three of these genes were predicted to express in hypodermis. We chose to focus on hypodermis since, to our knowledge, no large-scale study investigating hypodermal expression has been reported. Promoter-GFP constructs of two of the predicted hypodermal genes, K08B12.1 and F58H1.2, were most prominently expressed in the hypodermis at earlier stages (Figure 3A and 3B and Figure S4). The third gene, F55H12.4, showed strongest hypodermal expression during L4 and adult stages (Figure 3C). We also verified the expression of genes that we predicted to be expressed in muscle (C29F5.1, Figure 3D), intestine (F13D12.6, Figure 3E), and neurons (*gnrr-1*, Figure 3F). The tissue specific expression of *gnrr-1*, a homolog of the human gonadotropin releasing receptor, was previously studied using antibody staining [36]. While our algorithm predicted with high confidence that *gnrr-1* expresses in neurons, neuronal expression was not reported in that study, and the gene was not included in the Von Stetina *et al.* list of neuronally-enriched genes [22]. Nevertheless, our promoter-GFP *(Pgnnr-1::gfp)* construct expressed primarily in head neurons and ventral cord neurons (Figure 3D), validating our prediction. It is likely that the protein product of *gnrr-1* is heavily post-translationally modified, as species of multiple molecular weights are observed [36]. Thus, it is possible that differences in such modification explain the discrepancy between our gene expression results and the previous antibody staining experiment, due to epitope differences. Furthermore, *gnrr-1* is strongly over-expressed in L1 and L2 larval stages in multiple developmental microarray time courses, which is the pattern observed for many neuronal genes

### Regulatory motifs associated with tissue-specific expression

**Figure 4:**
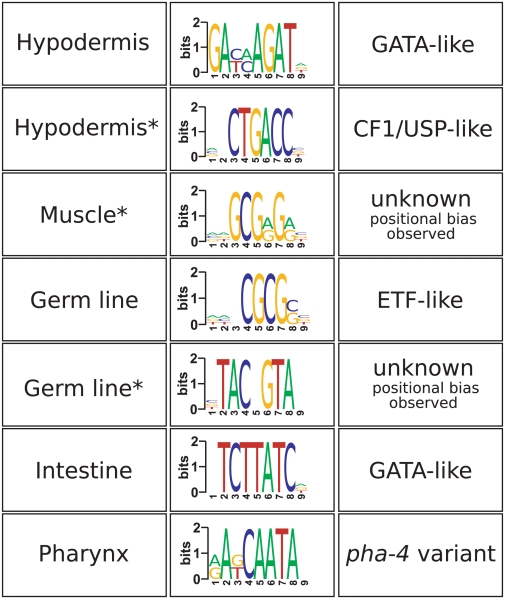
Motifs over-represented in the promoters of top predictions. (*) indicates motifs that have not been previously reported to be enriched in promoters of tissue-specific genes in *C. elegans*.

Our ability to make high-quality predictions also provided potential insights regarding the transcriptional regulation associated with the tissue-specific expression signal in whole animal data. We used a motif-finding program, FIRE [37], to identify motifs that are overrepresented in the upstream regions of our top-scoring predictions for each of the major tissues (Figure 4). While no genome-wide study of hypodermal expression has been published thus far, we were able to use our predictions to uncover motifs that are promising candidates for regulators of hypodermal transcription. A GATA-like motif was enriched among our top hypodermal predictions. This is consistent with previous studies showing that GATA transcription factors are essential for hypodermal cell specification, and that a GATA consensus sequence is required for hypodermal expression [38],[39]. In addition, we have identified a motif that is similar to the binding site for the CF1/USP-like nuclear hormone receptor that affects molting and developmental transitions in insects [40]. An intriguing possibility is that this motif and a functional USP homolog are involved in the nematode molting process as well, despite the fact that no direct USP homologs have been detected in the genome [41].

Using our germ-line predictions, we recovered an E2F-like motif (ETF). The *C. elegans* homolog of mammalian E2F, *efl-1*, is expressed exclusively in the germ line and is involved in oogenesis, regulating the expression of genes whose promoters contain the E2F binding motif [42]. Another motif, TAC.GTA, was also strongly represented among germ-line predictions. We could not detect a clear match to any known transcription factor consensus sequence, but a similar motif was previously discovered in a *C. elegans*-*C. briggsae* sequence comparison [43].

A GATA-like motif was also overrepresented among intestine predictions. GATA transcription factors are known to regulate expression of intestinal genes [44], and this motif is very similar to those reported by previous whole genome intestinal expression studies [17],[18] and aging studies [45],[46]. Our pharynx prediction yielded the largest number of motifs of any tissue. One of the motifs represents a possible match to the *pha-4* consensus ([T[AG]TT[TG][AG][TC] [15]) though other motifs did not resemble any known binding sites (see Table S2 for a complete list of motifs). Surprisingly, there was a shortage of neuronally-overrepresented motifs. In fact, the most significant result for neurons was instead motif avoidance. This is consistent with the hypothesis, supported by many experimental observations in *C. elegans* (see for example [47],[48]) that neuronal differentiation is a “ground state” that is superseded in non-neuronal cells.

### Identification of miRNA target tissue bias

The identification of global tissue-specific expression patterns allows us to address biological questions that are difficult to address experimentally, such as the question of tissue bias in microRNA targets. Non-coding microRNAs have emerged as critical developmental regulators, and are predicted to regulate the expression of a large fraction of all mammalian genes [49],[50]. Specific miRNAs direct development in particular tissues [51],[52], yet experimental identification of miRNA targets in individual tissues remains difficult. This is in part because expression of miRNA targets may be unchanged if translational inhibition, as opposed to mRNA degradation, is involved. Moreover, the ability to identify all targets for all miRNAs simultaneously is still more challenging.

**Figure 5:**
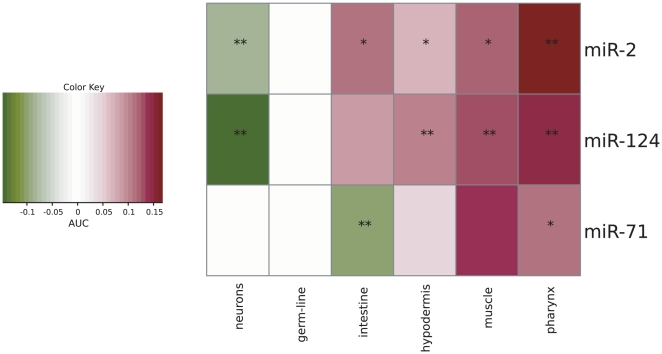
Tissue bias in microRNA target predictions. Our tissue-specific expression predictions allow us to systematically evaluate which *C. elegans* microRNA genes have a tissue bias in their predicted targets and thus are candidates for regulating tissue specific processes. For each microRNA gene we evaluate list of potential targets (as generated by three target prediction algorithms) against our tissue expression prediction scores using a rank test. (Average AUC is plotted, (*) indicates the interaction was significant (p<0.01) based on two out of three target sets, (**) was significant in all three. For each microRNA-tissue pair, enrichment (red) signifies that the targets of that microRNA are predicted to express in that tissue with scores that are significantly higher than would be expected if no bias is present. Avoidance (green) signifies that the microRNA targets have expression prediction scores that are significantly lower than expected. Since microRNAs down-regulate the levels of their targets, avoidance in one tissue coupled with preference in several others may imply involvement in differentiation, whereby the microRNA downregulates alternative tissue expression profiles.

Previous studies using human data have detected cell type-specific signatures among miRNA targets [53]. To address this problem in *C. elegans*, we leveraged our predictions of tissue-specific expression to investigate tissue bias, as measured by a rank-based statistic, among a list of likely *C. elegans* miRNA targets predicted by Miranda [54], TargetScan [49], and PicTar [55]. While many miRNAs had no detectable tissue bias among their targets, a subset showed significant tissue preference or tissue avoidance (see Figure S3 for all microRNAs-tissue interactions). In particular, robust tissue avoidance for three microRNAs was detected in all three sets of target predictions (Figure 5). The *miR-124* mammalian homolog is known to induce neuronal differentiation [52]. Our analysis demonstrates that its predicted targets are depleted for neuronal genes, while enriched for genes specific to other somatic tissues; these results suggest that its function is conserved in *C. elegans*. *miR-2* showed a pattern of neuronal depletion similar to *mir-124's* pattern, implying that it is also involved in neuronal differentiation; this is consistent with the exclusively neuronal pattern of GFP expressed from the *miR-2* promoter [56]. The *mir-71* target set, on the other hand, is significantly depleted for intestinal genes but enriched for genes expressed in muscle, hypodermis and pharynx. In contrast to *miR-2*, the anatomical expression of *miR-71* appears to be ubiquitous, suggesting that tissue-centric target analysis provides complementary information that is not captured by expression studies.

### Exploration of tissue-specific function of genes through functional networks

We have been able to leverage diverse microarray data to predict tissue-specific expression, including for genes expressed in more than one tissue. However, many genes that are expressed in several tissues (or ubiquitously) perform different functions in different cellular contexts. A natural way to explore such functional roles is through functional interaction networks, which connect genes that participate in the same biological process, an approach that has been used by us and others to examine functional roles of proteins on whole-genome scale [32],[57]. In contrast to previous approaches, in the case of tissue-specific functional networks, a network for a given set of genes may vary depending on the tissue of interest, as the same set of gene products may not perform the same function or share the same physical or other interactions in different tissues.

We have developed an SVM-based algorithm to predict tissue-specific functional networks from our compendium of *C. elegans* transcriptional data. Although simple expression correlation has often been used to investigate gene function on a global (non tissue-specific) level, our analysis above (and in Figure S1) demonstrates that a single global correlation computation is unable to distinguish between tissue-specific effects. On the other hand, the observation that whole-animal microarrays may contain a strong tissue specific signal suggests that it is possible to assess the tissue-dependent functional roles of genes given the right analytic approach. Thus, we have developed a network generation algorithm in which certain experiments are trusted more or less depending on the extent to which they reflect a particular tissue-specific functional signal.

Similarly to previous network integrations, we define a gold standard of functional interactions that is then used to determine how data is combined into a network. However, in contrast to previous studies [32],[58], we define several tissue-specific gold standards, one for each tissue, and we use an SVM rather than a Bayesian formulation to combine microarray data. An advantage of the SVM for this problem is that SVMs have the ability to adjust weights of individual experiments while Bayesian integration typically assigns weights to whole datasets. In the case of the *C. elegans* compendium, the ability to treat each experiment individually is crucial for prediction of tissue-specific networks, as a single dataset can contain experiments that are informative for different tissues. For example, within a single developmental time course (see Figure 1B), early larval stages are informative of neurons, when neuronal cells are overrepresented, while the adult stage is highly informative of germ-line.

**Figure 6:**
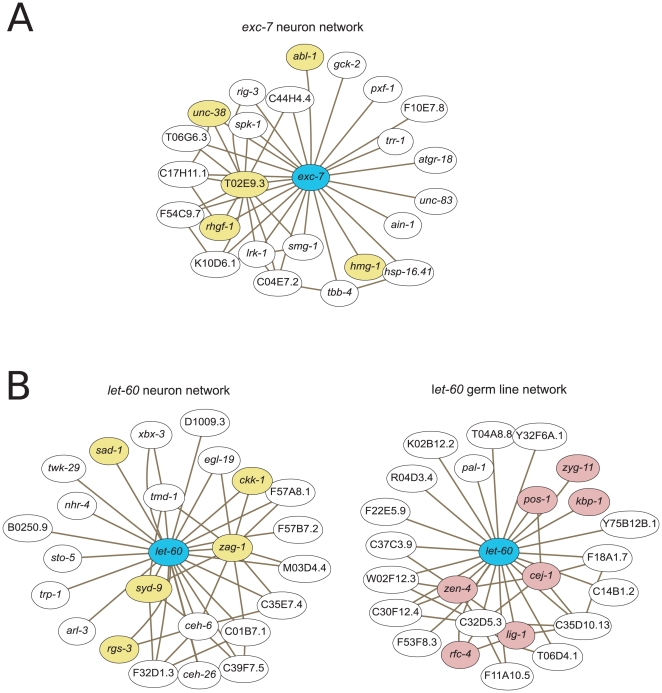
Tissue-specific weighted correlation networks allow elucidation of tissue-specific gene function. Top 20 predicted interactions partners and strongest inter-partner interactions are shown. Genes are colored according to known tissue-specific function: yellow indicates neuronal function, and red indicates involvement in a germ-line/oocyte process. (A) Neuron-specific network around *exc-7*. An extended SVM algorithm was used to predict tissue-specific functional interactions. Although *exc-7* is best characterized as playing a role in the formation of the excretory cell, it has also been shown to regulate cholinergic synaptic transmission. Many of its functional interaction partners are consistent with this neuron-specific function. (B)Tissue-specific networks for *let-60*. *let-60* is the homolog of mammalian Ras protein that is involved, among other processes, in chemosensation and progression through meiosis during oogenesis. The functional interaction partners identified for *let-60* are completely different in the neuron and germ line networks, reflecting that this gene plays a different functional role in the context of different tissues.

Using an SVM-based approach, we are able to integrate microarray data into different tissue-specific functional interaction networks. Such networks link genes that are likely to participate in the same process within a specific tissue context and contain information that may otherwise be overwhelmed in a global view of co-expression. As an example, we considered *exc-7*, an RNA-binding protein that is involved in the formation of the excretory canal, but that also plays a role in neuronal development, affecting cholinergic synaptic transmission [59]. Several of the interaction partners present in its neuron-specific interaction network support our understanding of *exc-7* neuronal function (Figure 6A): *hmr-1* is required for the outgrowth of some motor neurons [60]; *unc-38* is an acetylcholine receptor [12]; and the mammalian homolog of *abl-1* is involved in post-synaptic acetylcholine receptor clustering [61]. Another partner, *rhgf-1*, a RhoGEF, is known to regulate neurotransmitter release at the neuromuscular junction [62]. Our network results also suggest an interaction between *exc-7* and *smg-1*, a key component of the nonsense-mediated mRNA decay pathway, and *spk-1*, which is involved in mRNA splicing [12]. The presence of RNA processing genes among the interaction partners is potentially related to *exc-7's* RNA-binding function. A standard correlation computation produces an entirely different, non-neuron-specific set of genes associated with *exc-7*, including aquaporins and a gene involved in excretory cell formation (Figure S2). Our technique, on the other hand, automatically identifies a subset of microarray experiments with strong neuronal signals, and thus we are able to uncover neuron-specific functional interactions that are not immediately visible in a global correlation network.

Apart from finding tissue-specific interactions that can be lost in a global view, as in the above example, tissue-specific networks have the potential to tease out how the same gene may perform different functions within different tissue contexts. The *C. elegans* homolog of Ras, *let-60*, is a canonical example of a ubiquitously-expressed gene that participates in diverse processes. For example, *let-60* promotes progression through meiosis during oogenesis [63] and affects olfaction in neurons [64]. To explore these tissue-specific functions of this gene, we queried our germ line and neuronal networks with *let-60*. Two of the genes in the neuronal network are involved in chemosensation (Figure 6B): *rgs-3* is a regulator of G-protein signaling required for normal response to a variety of sensory stimuli [65], and *ckk-1* is a CaM kinase kinase that regulates the expression of chemosensory receptor genes [66]. Other neighbors in the network are involved in further aspects of neuronal function: *zaf-1*, *syd-9*, and *sad-1* function in synapse development, and *egl-19* is a calcium channel that contributes to fate specification in olfactory neurons [67].

By contrast, the germ line *let-60* network is comprised of an entirely different set of genes that are consistent with *let-60*'s function in meiosis: *cej-1(cpg-1)* is required for proper meiotic chromosome segregation [68], and *zyg-11* is part of a ubiquitin-ligase complex that promotes meiotic anaphase II [69]. Other interactors are likely to participate in related processes: *zen-4* is a kinesin protein that localizes to midzone microtubules [70]; *kbp-1* localizes to kinetochores [71]; and both *rfc-4* and *pos-1* affect a large number of events in the oocyte to embryo transitions [72]. Our networks focus on interaction information within a tissue-specific context, providing a framework for generating precise hypotheses about tissue-specific gene functions that can help direct follow-up experiments.

## Discussion

We have developed a computational method that accurately predicts tissue-specific expression based on expression profiles of primarily whole-animal microarrays. We show that strong tissue biases can be observed in data from microarray experiments, despite the fact that most *C. elegans* microarray experiments isolate mRNA from the whole animal, with the resulting expression values representing a population average of many cell types. With our SVM classifier, we were able to leverage these signals in existing whole-animal microarrays to produce predictions of tissue-specific gene expression and generate networks of tissue-specific functional interactions.

In addition to achieving accuracy higher than most directed microarray studies, our algorithm captures information about tissue-specific expression that is complementary to standard approaches. Microarray experiments analyzing tissue-specific expression are able to discover tissue-specific genes based on the difference in mRNA levels, a method that is ultimately sensitive to total mRNA abundance. Our method instead relies on co-expression with known tissue-specific genes in some informative condition, and thus identifies tissue-specific expression even for genes that have very low levels of expression in any one experiment. As we analyze microarray experiments from a variety of conditions, our approach can uncover genes expressed in a particular tissue in a condition-dependent manner which may be difficult to directly detect experimentally. For example, a promoter-GFP tagging study reported expression of *ins-7* exclusively in neurons [73], while our method predicts expression in both neuron and intestine. In fact, a recent study has shown that *ins-7* is indeed expressed in the intestine at a low level, with expression increasing significantly in aging animals and under conditions of high insulin signaling [74]. The earlier GFP study focused on young wild-type adults and thus did not identify this age-related expression. Thus, our method provides a valuable tool for study of tissue-specific expression that is relatively unbiased, as it does not rely on mRNA abundance directly and can leverage existing whole-animal compendia that provide a variety of developmental stages and conditions represented in these collections.

From a more general perspective, our method extracts tissue-specific expression and interaction information from large compendia of diverse microarray studies. Even in the case of larger animals where it may be feasible to perform microarray studies on dissected tissues, the underlying samples are nevertheless typically comprised of multiple cell types; a method to predict gene expression in tissue subtypes will be applicable to other organisms, limited only by the existence of an appropriate “gold standard” gene expression set. Our results demonstrate that sample heterogeneity, when appropriately analyzed, can provide valuable information regarding cell-type specific gene expression and function.

## Methods

### Gold Standard construction

Tissue localization data was retrieved from WormBase 170 [12] and parsed in a semi-automated way. Since a variety of terms are used to describe the same tissue and/or organ, we hand-compiled a table of tissue synonyms. In addition we applied some hierarchical propagation to tissue labels, such as assigning specific neurons to their neuron class (sensory, motor, interneurons). A majority of genes were reported to express in multiple tissues and each gene was considered a positive example for all tissue where it was found to express. This data includes all 1,872 genes investigated by the *C. elegans* Tissue Expression Consortium [8],[24] as well as expression patterns from smaller scale experiments, for a total of 2872 genes in the gold standard. These data did not include any large-scale expression studies (microarray or SAGE), and was limited to single-gene GFP or *in situ* experiments.

### Microarray data retrieval and formatting

We collected microarray data from 53 publications (see Supplementary website for complete list). The microarray values from a single publication were considered a coherent dataset and processed together. Data for single-channel platforms was transformed by dividing every gene value by its average over the dataset and taking the log of the result. All missing values were imputed using the KNN impute algorithm [75] (k = 10). For input to SVM learning the gene values within a single dataset were normalized to mean 0 and variance 1 before all datasets were concatenated. Since the SVM algorithm does not accommodate missing values, genes that were present in some datasets but not others were assigned a value of 0 when absent.

### Tissue bias in microarray experiments

For each tissue we used our gold standard to assign genes with known expression into 2 classes (tissue expressed and not tissue expressed). We the used the two classes and the microarray expression values to calculate an AUC score and the associated probability. The probabilities were used to correct the results for multiple hypothesis testing at a false discovery rate of 0.05.

### Single gene predictions

Single gene predictions were made using linear support vector machines (SVM). Given a set of genes known to be expressed in a particular tissue, the SVM identifies specific patterns of gene expression in a subset of experiments that differentiates these genes from those not expressed in the tissue. We performed 5-fold cross validation and optimized the parameters for maximal precision at 30% recall (fraction of genes in the gold standard correctly recalled) for major tissues and 10% recall for small tissues. SVMs are a maximal margin classifier that optimizes classification performance on the training set while maximizing model generalizing power by maximizing the distance of the nearest correctly classified examples to the separating plane. If 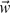 and 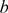 define the plane that separates the positive and negative examples, 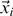 are the vectors of microarray data, 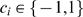 are the training labels, and 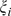 denote the degree of misclassification for each example, the SVM problem is to minimize
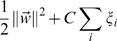
subject to 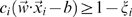. The constant 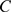 is empirically optimized to achieve the best performance at classifying new examples.

### GFP-promoter lines

Genes were selected based on the following criteria: top prediction scores that are specific to a single tissue, no previously reported tissue-specific localization to that tissue, and absence of any tissue-bias that could be inferred from sequence information alone. In particular, we avoided all collagen-related genes predicted to express in hypodermis due to ease of prediction of this particular tissue-specific expression from sequence. In addition, we specifically selected *gnrr-1* because of the discrepancy between our predictions (made with a top prediction score) and previously published results ([36]). Based on the above criteria we picked 14 genes, for which we obtained 9 lines; 6 of these fluoresced and these 6 are all shown in Figure 3. The GFP-promoter constructs were made using the Gateway system with the *unc-119* rescue plasmid pDestDD03 and promoter clones from the *C. elegans* promoterome [10]. The resulting constructs were bombarded into *unc-119(ed3)* mutants.

### Motif discovery

Motif discovery was performed for each tissue separately. For a single tissue, all the genes that were present in our microarray compendium were assigned a cluster number of 1 if they were in the top 500 predicted genes and a cluster number of 0 otherwise. This cluster assignment was used as input to the FIRE algorithm. Kmer length was set to 9 and default values were used for all other parameters.

### Network predictions

To generate the tissue-specific interaction standard we first generated a global functional interaction standard using a combination of GO, KEGG, and Textpresso-curated interactions [12],[76]. We then defined a set of tissue-specific interactions by cross-referencing with our gold standard of tissue expression used for single gene expression prediction. A tissue-specific interaction was defined as a pair of genes that were co-annotated to a specific GO term (see Supplementary methods) and were also both found to express in a particular tissue in our expression gold standard. The negative set was composed of positive interactions from other major tissues as well as random pairs of GO annotated genes. The classification problem is then to differentiate interactions specific to a particular tissue from interactions in other tissues as well as non-interacting gene pairs.

The algorithm computes a weighted sum of single experiment similarity measures. Since the expression values are normalized to have mean 0 and variance 1, single experiment similarity measures are thus single terms within a per-dataset Pearson correlation. The contribution of expression data to the final value is thus
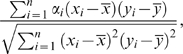
where 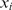 and 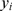 represent the expression values of genes 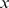 and 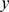 in experiment 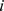 and 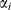 is the weight assigned to that experiment by the SVM classifier. (See Text S1 for a detailed description).

## Supporting Information

Figure S1 Method flow for SVM predictions. Five-fold cross validation was used to generate precision-recall plots, optimize learning parameters, and calculate estimated precision for novel predictions.
(0.22 MB PDF)

Figure S2 All miRNA target tissue interactions as measured by a ranksum statistic significant at 0.01. Numbers inside the cells represent how many target prediction sets gave a significant result (out of 3, Mirna, Pictar, Targetscan). When multiple target sets give significant results the ranksum statistic is averaged. “D” signifies that multiple target prediction sets gave significant results but disagreed in the direction.
(0.04 MB PDF)

Figure S3 A correlation network for exc-7 computed across the same expression data as was used for SVM learning. In contrast to the neuron-specific network generated by our method, this network is more representative of exc-7's excretory cell function. aqp-3 and aqp-10 are aquaporins, while eor-1 is known to affect excretory system development.
(0.03 MB EPS)

Figure S4 Additional imaged of strains expressing hypodermal GFP.
(0.07 MB PDF)

Table S1 Comparison of area under precision-recall curve (corrected for base-line) between a correlation-based method (sum of correlations with known tissue-specific genes) and an SVM based method.
(0.01 MB PDF)

Table S2 All significant motifs. Top 500 predictions for each tissue were used do define a cluster for FIRE motif analysis [1]. The regular expressions corresponding to significantly enriched or depleted motifs are shown.
(0.02 MB PDF)

Text S1 Network Prediction Methods
(0.03 MB PDF)
