# Supplementary material for: Global Prediction of Tissue-Specific Gene Expression and Context-Dependent Gene Networks in Caenorhabditis elegans
Source: PLoS Comput Biol. 2009 Jun 19;5(6):e1000417. doi: 10.1371/journal.pcbi.1000417 (PMC2692103; doi:10.1371/journal.pcbi.1000417)
Supplement: Figure S1 — Method flow for SVM predictions. Five-fold cross validation was used to generate precision-recall plots, optimize learning parameters, and calculate estimated precision for novel predictions. (0.22 MB PDF) [file pcbi.1000417.s001.pdf]

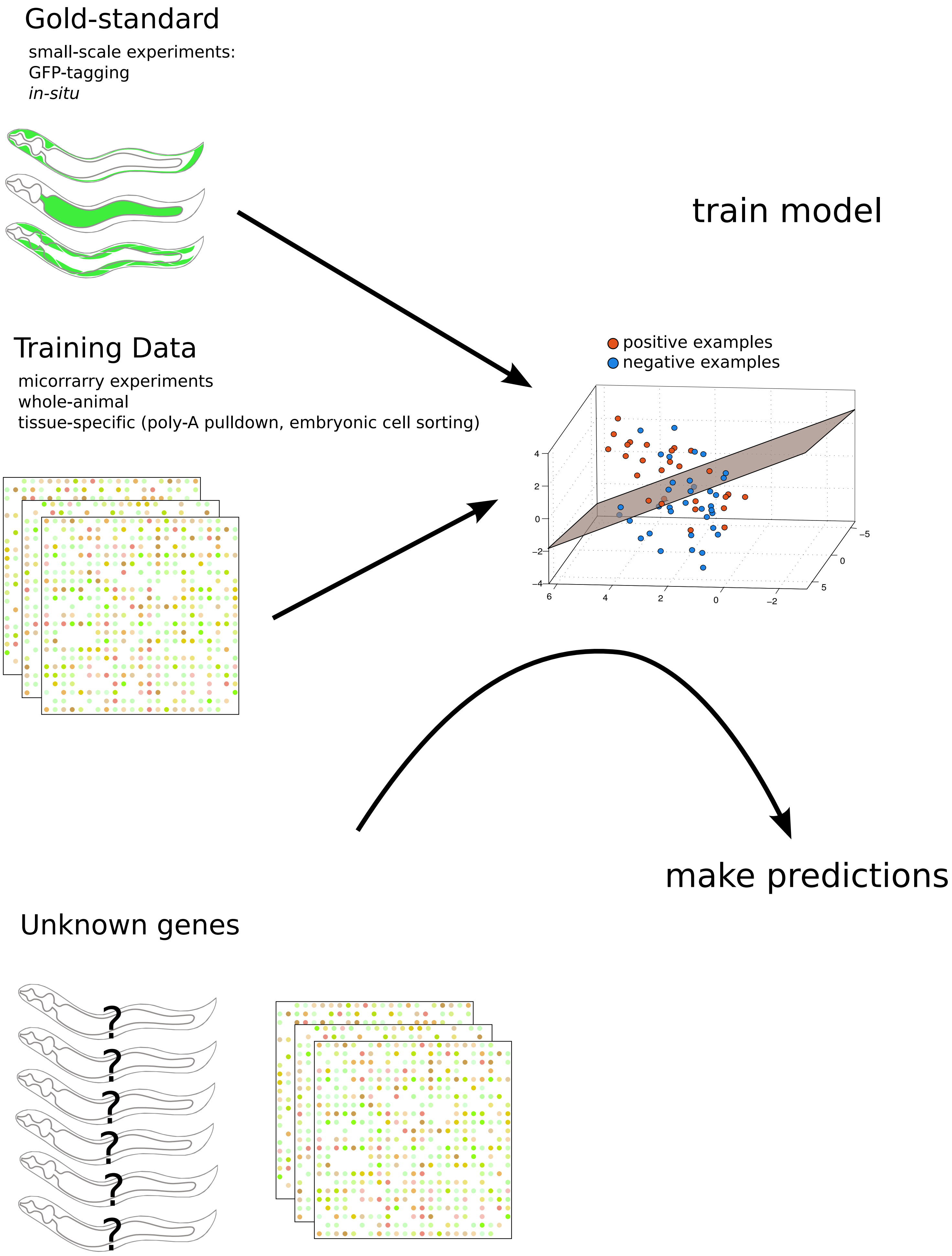
Gold-standard
small-scale experiments:
GFP-tagging
in-situ
train model
positive examples
negative examples
Training Data
micorrarry experiments
whole-animal
tissue-specific (poly-A pulldown, embryonic cell sorting)
make predictions
Unknown genes
