# Supplementary material for: Global Prediction of Tissue-Specific Gene Expression and Context-Dependent Gene Networks in Caenorhabditis elegans
Source: PLoS Comput Biol. 2009 Jun 19;5(6):e1000417. doi: 10.1371/journal.pcbi.1000417 (PMC2692103; doi:10.1371/journal.pcbi.1000417)
Supplement: Figure S2 — All miRNA target tissue interactions as measured by a ranksum statistic significant at 0.01. Numbers inside the cells represent how many target prediction sets gave a significant result (out of 3, Mirna, Pictar, Targetscan). When multiple target sets give significant results the ranksum statistic is averaged. “D” signifies that multiple target prediction sets gave significant results but disagreed in the direction. (0.04 MB PDF) [file pcbi.1000417.s002.pdf]

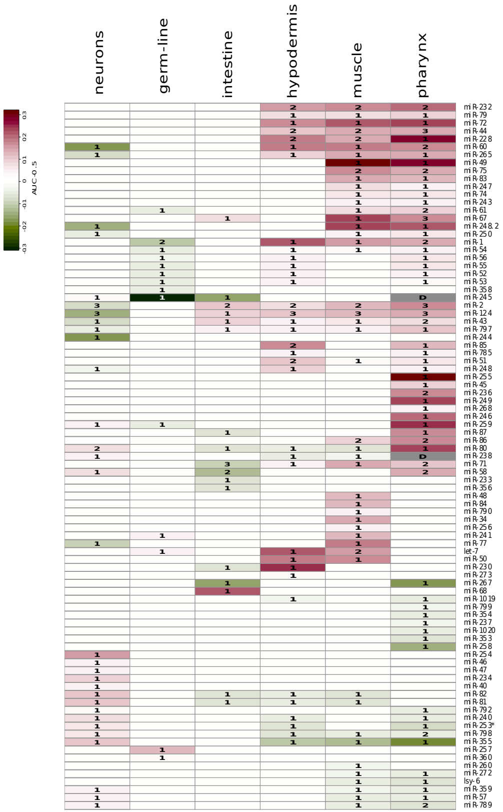
neurons
germ-line
intestine
hypodermis
muscle
pharynx
AUC-0.5
0.3
0.2
0.1
0
-0.1
-0.2
-0.3
miR-232
miR-79
miR-72
miR-44
miR-228
miR-60
miR-265
miR-49
miR-75
miR-83
miR-247
miR-74
miR-243
miR-61
miR-67
miR-248.2
miR-250
miR-1
miR-54
miR-56
miR-55
miR-52
miR-53
miR-358
miR-245
miR-2
miR-124
miR-43
miR-797
miR-244
miR-85
miR-785
miR-51
miR-248
miR-255
miR-45
miR-236
miR-249
miR-268
miR-246
miR-259
miR-87
miR-86
miR-80
miR-238
miR-71
miR-58
miR-233
miR-356
miR-48
miR-84
miR-790
miR-34
miR-256
miR-241
miR-77
let-7
miR-50
miR-230
miR-273
miR-267
miR-68
miR-1019
miR-799
miR-354
miR-237
miR-1020
miR-353
miR-258
miR-254
miR-46
miR-47
miR-234
miR-40
miR-82
miR-81
miR-792
miR-240
miR-253*
miR-798
miR-355
miR-257
miR-360
miR-260
miR-272
lsy-6
miR-359
miR-57
miR-789
