# Supplementary figures and images for: Global Prediction of Tissue-Specific Gene Expression and Context-Dependent Gene Networks in Caenorhabditis elegans
Source: PLoS Comput Biol. 2009 Jun 19;5(6):e1000417. doi: 10.1371/journal.pcbi.1000417 (PMC2692103; doi:10.1371/journal.pcbi.1000417)

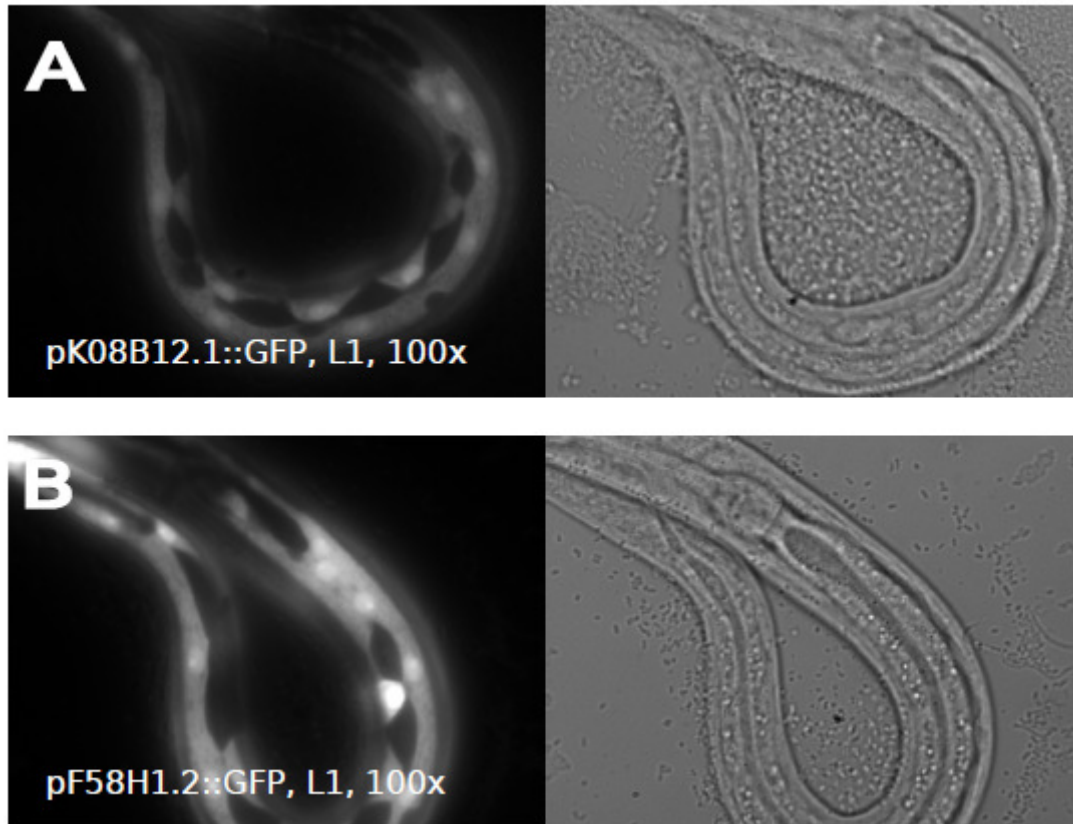

Additional imaged of strains expressing hypodermal GFP.

Supplement: Figure S4 — Additional imaged of strains expressing hypodermal GFP. (0.07 MB PDF) [file pcbi.1000417.s004.pdf]
