# Supplementary material for: Global Prediction of Tissue-Specific Gene Expression and Context-Dependent Gene Networks in Caenorhabditis elegans
Source: PLoS Comput Biol. 2009 Jun 19;5(6):e1000417. doi: 10.1371/journal.pcbi.1000417 (PMC2692103; doi:10.1371/journal.pcbi.1000417)
Supplement: Table S1 — Comparison of area under precision-recall curve (corrected for base-line) between a correlation-based method (sum of correlations with known tissue-specific genes) and an SVM based method. (0.01 MB PDF) [file pcbi.1000417.s005.pdf]

	Correlation	SVM	Fraction Improvement
germ line	0.12	0.23	1.92
neurons	0.05	0.16	3.14
intestine	0.06	0.18	3.10
muscle	0.12	0.23	1.92
hypodermis	0.09	0.13	1.41
pharynx	0.15	0.20	1.33

Table S1. Comparison of area under precision-recall curve (corrected for base-line) between a correlation-based method (sum of correlations with known tissue-specific genes) and an SVM based method.
