# Supplementary material for: Global Prediction of Tissue-Specific Gene Expression and Context-Dependent Gene Networks in Caenorhabditis elegans
Source: PLoS Comput Biol. 2009 Jun 19;5(6):e1000417. doi: 10.1371/journal.pcbi.1000417 (PMC2692103; doi:10.1371/journal.pcbi.1000417)
Supplement: Table S2 — All significant motifs. Top 500 predictions for each tissue were used do define a cluster for FIRE motif analysis [1]. The regular expressions corresponding to significantly enriched or depleted motifs are shown. (0.02 MB PDF) [file pcbi.1000417.s006.pdf]

germ-line

enriched

[CGT]TAC.GTA.
[AGT][ACT].CGCG[CG][CGT]
[CT][ACG]TTCGA[AG][AGT]
[AGT][AC]CCTGCG[ACT]

depleted

[ACG]TA[GT][AG]G[AT]C[AT]
[CGT]CTTA[CT]C[AT].

hypodermis

enriched

[ACG].CTGACC[CGT]
[CGT]A[CT][ACG][CG]GCA[AGT]
[AGT]A[AG]AGAG[AG][CGT]
[ACT]A[AG]GG[GT][CG]G.
GA[CT][AC]AGAT[AGT]

depleted

[AGT]ACTGAAA[ACT]muscle

intestine

enriched

.A[GT][ACT]GTCA[CT]
[AGT]TACA[CG][CG]C[CGT]

depleted

.[CT]CCCGCC[ACG]

muscle

enriched

[CGT]C[CGT][GT]CGT[AG][GT]
[ACG][AGT]GCG[AG]G[AG][CGT]

neurons

enriched

[ACT][GT]ACGT[AC][AT][AGT]
[ACG]AA[ACG][AG]GG[GT]G

depleted

.ACTGT[AG][CT].
[AG].[AGT]GTCGG[AC]
[ACG][AG]TCG[ACT]A[GT][GT]
[CT][CGT][CG]TTA[CT]C[AG]
.CG[AC]GA[CT]C.

pharynx

enriched

[ACT]ATT[GT]CGA[AGT]
.[CGT].CGCTC[CGT]
[AG]AG[AG]AA[AC]G[ACT]
[AGT][CT]GCG[GT]AC[ACT]
.AATGGAT[AGT]
[AGT][AC]TCAATA.
[CGT][CG]GTGCAC[AGT]

Table S2. All significant motifs. Top 500 predictions for each tissue were used do define a cluster for FIRE motif analysis[1]. The regular expressions corresponding to significantly enriched or depleted motifs are shown.

1. Elemento O, Slonim N, Tavazoie S (2007) A universal framework for regulatory element discovery across all genomes and data types. Mol Cell 28: 337-350.
