# Supplementary material for: Global Prediction of Tissue-Specific Gene Expression and Context-Dependent Gene Networks in Caenorhabditis elegans
Source: PLoS Comput Biol. 2009 Jun 19;5(6):e1000417. doi: 10.1371/journal.pcbi.1000417 (PMC2692103; doi:10.1371/journal.pcbi.1000417)
Supplement: Text S1 — Network Prediction Methods (0.03 MB PDF) [file pcbi.1000417.s007.pdf]

Network Predictions Methods

Data

Network predictions used the same expression data collection (including data processing) as the data used for single gene predictions (and described in the corresponding methods section). That is, all values were normalized to have a mean of 0 and a variance of 1 in a single dataset (a set of related experiments from a single publication).

Generating a Standard of tissue-specific Positive Interactions

To generate the tissue-specific interaction standard we first generated a global functional interaction standard using a combination of GO, KEGG, and curated interactions. All genes belonging to the same KEGG pathway were considered related. We applied a two step filter to the GO terms used for the standard. Firstly, we recruited biology researchers to hand curate a list of GO terms that they though were specific enough to be tested with a concrete experiment (see [1] for description of a similar process for the yeast annotations). In addition, we also removed all GO terms with size larger than 300 annotations to exclude terms that are too general. All genes that were annotated to the same GO term in the filtered set were then considered functionally related. We then defined a set of tissue-specific interactions by cross-referencing this set of functionally related genes with our gold standard of tissue expression. In order to be considered a tissue-specific interaction both genes had to express in that particular tissue and also be part of the set of functionally related genes.

Generating a Standard of Negative Interactions

Since we wanted to distinguish tissue-specific functional relationships both from non-tissue-specific functional interactions as well as non-interacting gene pairs, both kinds of negative examples were included. Specifically, all interactions from tissues other than the tissue in question were considered as negative examples. In addition, an equal number of random gene pairs that had GO annotations but were not considered functionally related by the criteria described above were added.

SVM Training

In order to get an interaction score for a specific gene pair both of the genes in that pair were held out in the training step. This avoids gene-level over-fitting, as otherwise some genes have a large number of interactions in the standard and the SVM algorithm may learn to predict pairs containing that gene as positives. The SVM constant C was optimized empirically though it had only a small effect on performance in the range that we explored.

The input to the SVM algorithm consisted of a vector for each gene pair, whose length was equal to the number of microarray experiments in our collection. Each entry in the vector is the product of the two normalized expression values for the two genes in that experiment. Since the expression values are normalized to have mean 0 and variance 1, single experiment similarity measures are thus single terms within a per-dataset Pearson correlation. The contribution of expression data to the final value is thus

$$\frac{\sum_{i=1}^{n} \alpha_i (x_i - \bar{x})(y_i - \bar{y})}{\sqrt{\sum_{i=1}^{n} (x_i - \bar{x})^2 (y_i - \bar{y})^2}},$$

where x_i and y_i represent the expression values of genes x and y in experiment i and α_i is the weight assigned to that experiment by the SVM classifier.

1. Myers CL, Barrett DR, Hibbs MA, Huttenhower C, Troyanskaya OG (2006) Finding function: evaluation methods for functional genomic data. BMC Genomics 7: 187.
